# Liver Fibrosis and Protection Mechanisms Action of Medicinal Plants Targeting Apoptosis of Hepatocytes and Hepatic Stellate Cells

**DOI:** 10.1155/2014/373295

**Published:** 2014-11-20

**Authors:** Florent Duval, Jorge E. Moreno-Cuevas, Maria Teresa González-Garza, Carlos Rodríguez-Montalvo, Delia Elva Cruz-Vega

**Affiliations:** ^1^Catedra de Terapia Celular, Escuela de Medicina, Tecnológico de Monterrey, Avenida Morones Prieto 3000 Pte., 64710 Monterrey, NL, Mexico; ^2^Centro de Enfermedades Hepáticas-Digestivas y Nutrición, Hospital San José, Avenida Morones Prieto 3000, 64710 Monterrey, NL, Mexico

## Abstract

Following chronic liver injury, hepatocytes undergo apoptosis leading to activation of hepatic stellate cells (HSC). Consequently, activated HSC proliferate and produce excessive extracellular matrix, responsible for the scar formation. The pandemic trend of obesity, combined with the high incidence of alcohol intake and viral hepatitis infections, highlights the urgent need to find accessible antifibrotic therapies. Treatment strategies should take into account the versatility of its pathogenesis and act on all the cell lines involved to reduce liver fibrosis. Medicinal plants are achieving popularity as antifibrotic agents, supported by their safety, cost-effectiveness, and versatility. This review will describe the role of hepatocytes and HSC in the pathogenesis of liver fibrosis and detail the mechanisms of modulation of apoptosis of both cell lines by twelve known hepatoprotective plants in order to reduce liver fibrosis.

## 1. Introduction

Fibrosis is an inappropriate tissue repair of the liver resulting from almost all of the chronic liver injuries including alcohol induced damage, chronic viral hepatitis, autoimmune, parasitic, and metabolic diseases and less frequent, toxic, or drugs exposure [1]. When fibrosis is not controlled, it can further progress into cirrhosis. In contrast with the traditional idea that cirrhosis is an irreversible state, there is solid evidence indicating that fibrosis even cirrhosis could be reversible [2].

Liver fibrosis is an important public health concern with significant morbidity and mortality [3]. Hundreds of millions of people worldwide suffer from cirrhosis [4]. Chronic viral hepatitis B and C, alcoholic liver diseases, and nonalcoholic fatty liver diseases are the three most common causes [5]. Prevalence of chronic liver diseases, hence hepatic fibrosis-cirrhosis, is predicted to increase, due in part to the rising prevalence of obesity and metabolic syndrome, especially in developed countries [6].

Pathogenesis of liver fibrosis is complex and varies between the different kinds of hepatic injuries. Usually after an acute liver damage, parenchymal cells regenerate and replace the necrotic and apoptotic cells; this process is associated with an inflammatory response and a limited deposition of extracellular matrix (ECM). When the injury persists, eventually the regenerative response fails and the hepatocytes are substituted by abundant ECM mainly composed by collagen type I-III-IV, fibronectin, elastin, laminin, and proteoglycans. Hepatic stellate cells (HSC) are the main sources of ECM [7].

There is no standard treatment for liver fibrosis, although it is known that reducing liver injury events, such as interruption of alcohol intake or successful treatment of viral hepatitis, contributes to the control of the process. Nevertheless, these actions do not seem to be sufficient, in the vast majority of patients, to avoid progression to cirrhosis [8]. Even though important advances have been made in the knowledge of the pathogenesis of hepatic fibrosis for the past 20 years, there are still important gaps to translate this basic information into efficient antifibrotic drugs. Treatment strategies for liver fibrosis should take into account the versatility of its pathogenesis and acting on all the cell lines involved starting with HSC and hepatocytes.

**Figure 1:**
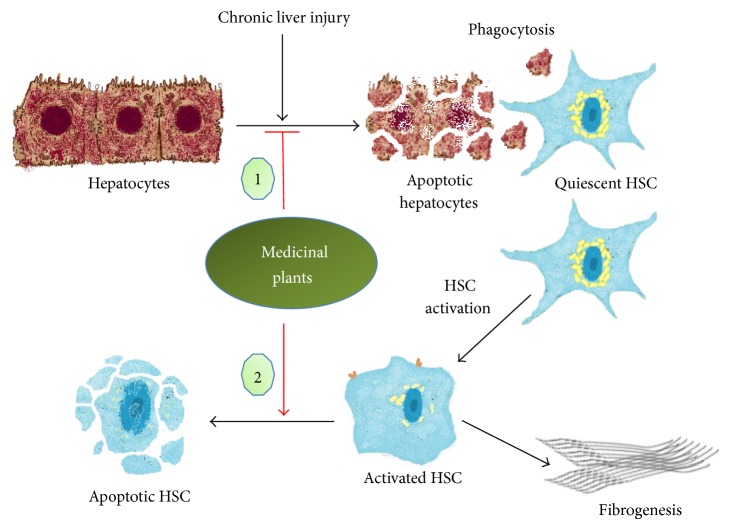
Anti-liver fibrosis of medicinal plants targeting apoptosis of hepatocytes and hepatic stellate cells. (1)* C. longa*,* S. marianum*,* G. biloba*,* S. miltiorrhiza*,* G. glabra*,* S. baicalensis*,* Phyllanthus* species,* B. aristata*,* P. kurroa*,* Ginseng* species,* A. paniculata*. (2)* C. longa*,* G. biloba*,* S. miltiorrhiza*,* G. glabra*,* S. baicalensis*,* B. falcatum*, and* Ginseng* species.

Supported by their safety, cost-effectiveness, and versatility, medicinal plants enjoy a growing popularity as antifibrotic agents. We already reviewed how medicinal plants reduce liver fibrosis by inhibiting HSC activation and reducing ECM deposition [9]. However, other antifibrotic mechanisms could explain this activity such as modulation of apoptosis of different cell lines. This review focuses on two more ways by which the bioactive compounds from twelve known hepatoprotective plants, including* Curcuma longa*,* Silybum marianum*,* Ginkgo biloba*,* Salvia miltiorrhiza*,* Glycyrrhiza glabra*,* Scutellaria baicalensis*,* Bupleurum falcatum*,* Phyllanthus* species,* Berberis aristata*,* Picrorhiza kurroa*,* Ginseng* species, and* Andrographis paniculata*, reduce liver fibrosis by targeting apoptosis: the induction of HSC apoptosis and the protection of hepatocytes from apoptosis (Figure 1).

## 2. Induction of HSC Apoptosis

### 2.1. Role of HSC in the Pathogenesis of Liver Fibrosis

Quiescent HSC act as the major vitamin A-storing cells located in the perisinusoidal space of Disse between the basolateral surface of hepatocytes and the antiluminal side of sinusoidal endothelial cells [10]. HSC are the key effectors in the development of liver fibrosis.

The process of liver fibrosis initiates with HSC activation; this is mainly due to several mediators' effects, like reactive oxygen species (ROS), lipid peroxidation (LPO) products, and fibrogenic cytokines such as transforming growth factor beta (TGF-*β*1) and platelet derived growth factor (PDGF). These substances come from damaged hepatocytes, as we detailed below, and/or activated Kupffer cells, macrophages, and platelets following hepatic injury [11, 12]. Activated HSC acquire different phenotypes such as enhanced production of ECM, expression of contractile smooth muscle *α*-actin (*α*-SMA), enhanced proliferation, secretion of pro-inflammatory cytokines, and release of matrix-degrading enzymes and their inhibitors [13]. Activated HSC remain the main contributors of major and minor matrix proteins of the fibrotic liver including types I, III, and IV collagens, fibronectin, laminin, and proteoglycans [11, 14] even though many other cells, including portal fibroblasts, circulating cells from the bone marrow, hepatocytes, and biliary epithelial cells that undergo an epithelial to mesenchymal transition, also produce ECM [15].

Activated HSC are also characterized by an enhanced survival. Hepatic macrophages promote the survival of activated HSC in a nuclear factor-kappaB- (NF-*κ*B-) dependent manner and thereby promote liver fibrosis [16]. However, inhibition of NF-*κ*B pathway reverses hepatic fibrosis by stimulating HSC apoptosis [17], thereby highlighting selective induction of HSC apoptosis as a promising strategy to treat liver fibrosis [4, 18–21].

### 2.2. Hepatic Stellate Cells as Targets of Antifibrotic Medicinal Plants

Twenty-three articles were chosen (Table 1). Curcumin from* C. longa* and bioactive compounds from* S. miltiorrhiza*, including IH764-3, tanshinones I and IIA, and salvianolic acid A, are by far the most investigated, followed by compounds extracted from* Ginseng* species. Consequently, their mechanisms of induction of HSC apoptosis have been well characterized. On the opposite, apoptosis induction by* G. biloba* extract [22], 18*α*-glycyrrhizin from* G. glabra* [23], baicalin from* S. baicalensis*, and saponins from* B. falcatum* [24] have only been observed; thus, their mechanisms need to be clarified. No proof that* S. marianum*,* Phyllanthus* species,* B. aristata*,* P. kurroa*, and* A. paniculata* produce compounds that induce HSC apoptosis exists. The apoptotic events induced by medicinal plants present similarities since they all act by modulating mitochondrial caspases cascade. However, different targets have been identified upstream to the apoptotic cascade.

Curcumin increases and decreases Bcl-2-associated X protein (Bax) and B-cell lymphoma 2 (Bcl-2) expressions, respectively [25, 26, 28, 29], promotes cytochrome c release from mitochondria into cytoplasm [31], and increases caspase-3 activity [25, 26, 30] in primary cultured rat HSC. Induction of apoptosis by curcumin correlates with its inhibitory effect on NF-*κ*B [29], which involves the stimulation of gene expression of peroxisome proliferator-activated receptor gamma (PPAR*γ*) [25–29] by blocking TGF-*β*, PDGF, and epidermal growth factor (EGF) signaling pathways through interruption of extracellular signal-regulated kinases (ERK), c-Jun N-terminal kinases (JNK), and phosphatidylinositide 3-kinases/protein kinase B (PI-3K/AKT) pathways [28]. Additionally, modulation of BAX and cellular FADD-like IL-1*β*-converting enzyme- (FLICE-) like inhibitory protein (c-FLIP) (CASP8 and FADD-like apoptosis regulator) expressions and reduction of the expression of Wnt signaling pathway components, axis inhibition protein 2 (AXIN2), and FOS-like antigen 1 (FRA1) mediate induction of HSC apoptosis by curcumin in human telomerase reverse transcriptase HSC [32].

Root of* S. miltiorrhiza* promotes HSC apoptosis by increasing Bax and Fas expressions and decreasing B-cell lymphoma-extralarge (Bcl-XL) expression* in vitro* in HSC-T6 cells [41]. Monomer IH764-3, tanshinone I, tanshinone IIA, salvianolic acid A, and fraction PF2401-SF (tanshinone I, tanshinone IIA, and cryptotanshinone) mediate proapoptotic effects of* S. miltiorrhiza* root. Like curcumin, they act via increasing Bax/Bcl-2 ratio [37–40], decreasing mitochondrial membrane potential (MMP) [36, 37], inducing cytochrome c release [36–38], stimulating poly ADP ribose polymerase (PARP) cleavage [36–38, 40], and enhancing caspase-3 and 9 activities [33, 36–40]. IH764-3 downregulates the expression of focal adhesion kinase (FAK) and phosphorylated FAK, ERK, and phosphorylated ERK to promote HSC apoptosis [34, 35]. Tanshinone IIA acts by enhancing prohibitin expression, inducing intracellular translocation of the cytosolic C-Raf protein to the membrane, increasing p-ERK, and suppressing AKT phosphorylation, thereby indicating that tanshinone IIA induces apoptotic cell by promoting binding between prohibitin and C-Raf which in turn activates mitogen activated protein kinases (MAPK) pathway and consequently Bax/caspase cascade [38]. Interestingly, salvianolic acid A also reduces AKT phosphorylation [39].

Saponins from* P. notoginseng* induce HSC of apoptosis* in vitro* but their mechanisms have not been investigated [24]. 20-O-Beta-D-glucopyranosyl-20(S)-protopanaxadiol, a ginsenoside metabolite, triggers apoptosis in activated HSC by reducing MMP and increasing caspase-3 activity and PARP cleavage [43]. Moreover, 25-OCH3-PPD, a dammarane-type triterpene isolated from* P. notoginseng*, induces the apoptosis of HSC activated by tumor necrosis factor alpha (TNF-*α*). 25-OCH3-PPD increases the level of cleaved caspase-3, downregulates the ratio of Bcl-2/Bax, and the expression of caspase-3 inhibitor survivin. This effect takes place through increasing the expression of c-FLIPL and decreasing c-FLIPs and X-linked inhibitor of apoptosis protein (XIAP) expressions, which lead to NF-*κ*B activation via degradation and phosphorylation of inhibitor of NF-*κ*B alpha (I*κ*B*α*) and translocation of p65 subunit into the nucleus [44].

## 3. Protection of Hepatocytes from Apoptosis

### 3.1. Role of Hepatocytes in the Pathogenesis of Liver Fibrosis

Hepatocytes account for about 80% of the liver. Under chronic liver injury, hepatocytes undergo apoptosis liberating hepatocyte-derived apoptotic bodies [45]. This initial event is no longer viewed as a silent consequence of liver injury but rather as a potent inductor of liver fibrosis [46]. Profibrogenic response following hepatocytes apoptosis is enabled by the capacity of HSC to perform phagocytic function [46, 47]. Phagocytosis of the hepatocyte-derived apoptotic bodies directly induces HSC activation and matrix deposition as it up-regulates TGF-*β*1 and induces collagen *α*1(I) through PI-3K and p38MAPK pathways [46, 48, 49]. This profibrogenic event requires nicotinamide adenine dinucleotide phosphate reduced (NADPH) oxidase activation [46, 50]. Concurrently, an indirect signal mediated by the generation of damage-associated molecular patterns (DAMPs) results in HSC activation [49]. DNA from apoptotic hepatocytes induces HSC differentiation by upregulating TGF-*β*1 and collagen expression and inhibiting chemotaxis of HSC, so mobile HSC stop when they reach an area of apoptosing hepatocytes, via toll-like receptor 9 (TLR9) [51]. Adenosine, another product of apoptosing hepatocytes, has been identified also as a mediator of fibrogenic cascade [52]. In addition, phagocytosis of apoptotic bodies promotes HSC survival through Janus kinase/signal transducer and activator of transcription (JAK/STAT) and AKT/NF-*κ*B-dependent pathways, contributing to progression of liver fibrosis [53]. Thus therapeutic strategies, which aim to protect hepatocytes from apoptosis, could be useful to reverse liver fibrosis [54].

### 3.2. Hepatocytes as Targets of Antifibrotic Medicinal Plants

Thirty-two articles were selected (Table 2). It has been demonstrated that all the reviewed plants, except* B. falcatum*, produce compounds that inhibit apoptosis of hepatocytes induced by a wide range of agents, including ethanol, iron, carbon tetrachloride (CCl_4_), tert-butylhydroperoxide (t-BHP), toxic bile salts (glycochenodeoxycholic acid [GCDC]), thioacetamide (TAA), lipopolysaccharide (LPS) with D-galactosamine (D-GalN), concanavalin A (Con A), high free fatty acids (HFFAs), and so forth. Most investigated compounds are those of* C. longa*,* G. biloba*,* S. miltiorrhiza*, and* G. glabra*. Almost all the bioactive components from reviewed plants act similarly by inhibiting mitochondrial pathway of apoptosis and reducing oxidative stress.

Curcumin inhibits ethanol-, iron-, and HFFAs-induced apoptosis in primary cultured rat hepatocytes [55–57]. Curcumin regulates mitochondrial biogenesis [57], inhibits LPO [55] and ROS synthesis [56, 57], downregulates Bcl-2 and Bcl-XL expressions [56], inhibits cytochrome c release [55], restores MMP [57], and suppresses caspase-3 activity [56]. Cytoprotective effects of curcumin are mediated by downregulation of NF-*κ*B activity [56], especially p65 subunit [57].

Glycyrrhizin, also known as glycyrrhizin acid, is the main bioactive component from* G. glabra*. Glycyrrhizin protects hepatocytes from apoptosis* in vitro* and* in vivo*.* In vitro*, it prevents glutathione depletion, decreases ROS generation and LPO, and increases superoxide dismutase (SOD) activity, highlighting the importance of its antioxidant properties to inhibit hepatocytes apoptosis [70]. It also inhibits MMP, cytochrome c release, p38 activation, and caspases-3 and -9 activities [70, 75]. Additionally, it decreases nitric oxide (NO) and intercellular adhesion molecule 1 (ICAM-1) expression [72]. Involvement of caspases pathway inhibition has also been observed* in vivo* [71, 73]. Nevertheless, protection of hepatocytes could also occur through caspases-independent pathway related to the inhibition of the release of interleukin-18 [74].

*S. miltiorrhiza* bioactive components include tanshinones and salvianolic acids. PF2401, a standardized fraction of root of* S. miltiorrhiza*, and its components tanshinone I, tanshinone IIA, and cryptotanshinone, protect primary cultured rat hepatocytes from GCDC-, LPS-, and ethanol-induced apoptosis by inhibiting MAPK pathway via blockage of JNK and p38 phosphorylations [66], lipid accumulation, and activation and transactivation of genes involved in fatty acid biosynthesis through suppression of the nuclear translocation of sterol regulatory element binding protein-1 (SREBP-1) [67]. Besides, tanshinone IIA inhibits synthesis of ROS and reactive nitrogen species, fatty acid synthesis, and the opening of mitochondrial permeability transition and stimulates fatty acid oxidation by decreasing and increasing stearoyl-CoA desaturase-1 (SCD1) and retinoid-X receptor-alpha (RXR-*α*), respectively, in LPS-, ethanol-, and CCl4-treated primary cultured rat hepatocytes [62, 64].* In vivo*, its antiapoptotic properties have been related to the downregulation of insulin-like growth factor-binding protein 7 (IGFBP7) [63]. Finally, protection of hepatocytes from apoptosis by salvianolic acid B is associated with its ability to reduce the expression of tumor necrosis factor alpha receptor type 1 (TNFR1), balance the expression of Bcl-2 family members, decrease release of cytochrome c, and inhibit caspase-3* in vitro* and* in vivo* [69].

Bioactive extract of* G. biloba* (EGB) is composed of 6% of terpenes and 24% of flavonols heterosides. EGB inhibits technetium 99mTc-, ethanol-, and CCl_4_-induced apoptosis in rats principally by reducing oxidative stress via inhibiting LPO [59–61], glutathione depletion, promoting SOD, glutathione peroxidase (GPx), and catalase (CAT) activities and upregulating heme oxygenase-1 (HO-1) expression and activity [60]. It also reduces p53/Bcl-2 ratio [61].

## 4. Highlights

In this review, we highlighted the polyvalence of* C. longa*,* S. marianum*,* G. biloba*,* S. miltiorrhiza*,* G. glabra*,* S. baicalensis*,* B. falcatum*,* Phyllanthus* species,* B. aristata*,* P. kurroa*,* Ginseng* species, and* A. paniculata* and respective bioactive compounds and extracts to reduce liver fibrosis targeting apoptosis of hepatocytes and activated HSC. By protecting hepatocytes from apoptosis, medicinal plants are able to inhibit the liberation of hepatocyte-derived apoptotic bodies and DAMPs, some of the initial profibrogenic stimuli that converge to activation and survival of HSC, while inducing apoptosis of activated HSC; they eliminate the main source of ECM. Regulation of mitochondrial pathways of apoptosis by vegetal compounds mainly explains the induction and protection of apoptosis* in vitro* and* in vivo*.

To induce apoptosis of activated HSC, medicinal plants increase proapoptotic proteins, such as Bax and Fas, and decrease antiapoptotic proteins, like Bcl-2 and Bcl-xl. The increase in Bax/Bcl-2 ratio stimulates the release of cytochrome c from mitochondria into cytosol through MMP. The release activates initiator caspases (caspases-8 and -9) which leads to activation of executioner caspases such as caspase-3, responsible for the apoptotic process eventually through cleavage of PARP, a protein involved in repairing DNA damage. Opposite effects mediate the antiapoptotic properties of medicinal plants to protect hepatocytes.

NF-*κ*B, a transcription factor involved in inflammatory and apoptotic response, seems to play an intermediary role in the modulation of apoptosis of activated HSC and hepatocytes. Interestingly, inhibition of NF-*κ*B activity results in opposite effects in activated HSC and hepatocytes. Medicinal plants downregulate NF-*κ*B activity in activated HSC leading to inhibition of survival and promotion of apoptosis. On the contrary, inhibition of NF-*κ*B activity results in the protection from cell death in hepatocytes. Involvement of NF-*κ*B in both antifibrotic activities suggests a common stimulus of activation of this transcriptional factor between medicinal plants. Antioxidant properties of bioactive compounds from reviewed plants could explain such a similarity. Indeed, NF-*κ*B is regulated by the intracellular redox state thereby implying that antioxidant compounds of reviewed medicinal plants reduce chronic liver injury-induced oxidative stress which is sensed by NF-*κ*B resulting in modulation of apoptosis in hepatocytes and HSC [87].

Antiliver fibrosis mechanisms of medicinal plants have been mostly studied in liver fibrosis models* in vitro* and* in vivo*. Clinical studies are sparse and mainly use chronic hepatitis B and C patients to assess the hepatoprotective effects of medicinal plants. Consequently, more clinical investigations on fibrosis induced by other agents than HBV and HCV are urgently needed. Silymarin, glycyrrhizin, and* Salvia miltiorrhiza* have been more or less successfully tested. Glycyrrhizin could benefit patients with chronic hepatitis C nonresponders or unlikely respond to interferon therapy by decreasing alanine transaminase and improving necroinflammation [88–91]. Silymarin has also been tested in patients infected with HCV. However, contradictory results, as well as its low bioavailability, have not been able to conclude about its clinical efficacy [92–95]. Finally,* Salvia miltiorrhiza* injection and one of his bioactive compounds, salvianolic acid B, could be relevant in the treatment of hepatitis B [96–99].

Besides HSC and hepatocytes, inflammatory and immune cells take part actively in the fibrogenic response. In addition, important events, including HSC activation, ECM deposition, inflammation, and oxidative stress, are involved in the pathogenesis of liver fibrosis. Such targets could be relevant to reducing hepatic fibrosis. The extensive literature search made as part of this review evidenced other mechanisms besides the ones described here, by which medicinal plants reduce liver fibrosis, including previously reviewed inhibition of HSC activation and reduction of ECM deposition [9], as well as lowering of oxidative stress and suppression of inflammation and immune response.

## 5. Conclusion

Medicinal plants could be a source of polyvalent antiliver fibrosis compounds targeting apoptosis of hepatocytes and activated HSC. The importance of knowing the main mechanisms, by which medicinal plants act as antifibrotic agents, provides options for the development of pharmaceutical compounds and their subsequent use in medical practices.

## Figures and Tables

**Table 1:** Mechanisms of induction of hepatic stellate cells apoptosis by medicinal plants.

Plants	Bioactive compounds and/or extracts	Types of study	Cell lines/animals used (fibrogenic inducers)	Mechanisms of induction of HSC apoptosis
*C. longa *	Curcumin	I [25]	PCR-HSC	↑caspase-3, ↓Bcl-2, ↑PPAR-*γ* and ↓NF-*κ*B
		I [26]	PCR-HSC	↑PPAR-*γ*, ↑Bax, ↓Bcl-2 and ↑caspase-3
		I [27]	PCR-HSC	↑PPAR-*γ*
		I [28]	PCR-HSC	↑Bax, ↓Bcl-2, ↑PPAR-*γ*, ↓ERK, ↓JNK and ↓PI-3K/AKT
		I [29]	PCR-HSC	↑PPAR-*γ*, ↑Bax, ↓Bcl-2 and ↓NF-*κ*B p65
		I, II [30]	PCR-HSC and SD rats (CCl_4_)	↑caspase-3
		I [31]	HSC-T6 (TGF-*β*1)	↑cytochrome *c* release
		I [32]	Human telomerase reverse transcriptase HSC	Modulate BAX and FLIP and ↓Wnt signaling pathway components AXIN2 and FRA1

*S. miltiorrhiza *	IH764-3	I [33]	CFSC	↑caspase-3
		I [34]	HSC (H_2_O_2_)	↓ERK
		II [35]	SD rats (BDL)	↓FAK, ↓p-FAK, ↓ERK and ↓p-ERK
	Tanshinone I	I [36]	T-HSC/Cl-6	↑caspase-3, ↑PARP, ↑cytochrome *c* release and ↓MMP
	Tanshinone IIA	I [37]	T-HSC/Cl-6	↑caspase-3, ↑PARP, ↑cytochrome *c* release, ↑Bax, ↓Bcl-2 and ↓MMP
		I, II [38]	HSC-T6 and Wistar rats (DMN)	↑prohibitin, ↑C-Raf membrane tanslocation, ↑pERK, ↓AKT phosphorylation, ↑Bax, ↓Bcl-2, ↑cytochrome *c* release, ↑caspase-3, ↑caspase-9 and ↑PARP cleavage.
	Salvianolic acid A	I [39]	HSC-T6 (PDGF-BB)	↓AKT phosphorylation, ↑caspase-3 and ↓Bcl-2
	PF2401-SF	I, II [40]	T-HSC/Cl-6 and SD rats (CCl_4_)	↑caspase-3, ↑caspase-8, ↑caspase-9, ↑PARP cleavage, ↑Bax and ↓Bcl-2
	Root of *S. miltiorrhiza *	I [41]	HSC-T6	↑Bax, ↑Fas and ↓Bcl-XL

*G. glabra *	18*α*-glycyrrhizin	I, II [23]	CFSC and SD rats (CCl_4_)	↓NF-*κ*B

*B. falcatum *	Saikosaponin A and D	I [42]	HSC-T6	↓ERK

*P. notoginseng *	20-O-beta-D-glucopyranosyl-20(S)-protopanaxadiol	I [43]	T-HSC/Cl-6	↓MMP, ↑caspase-3 and ↑PARP cleavage
	25-OCH_3_-PPD	I [44]	T-HSC/Cl-6 (TNF-*α*)	↑caspase-3, ↓survivin, ↓Bcl-2, ↑c-FLIP_L_, ↓c-FLIP_S_, ↓XIAP, ↑NF-*κ*B p65 nuclear translocation and ↓I*κ*B-*α*

Abbreviations: ↑: inductor effect, ↓: inhibitor effect; I: *in vitro*; II: *in vivo*; AKT: protein kinase B; Bax: Bcl-2-associated X protein; Bcl-2: B-cell lymphoma 2; Bcl-XL: B-cell lymphoma-extralarge; BDL: bile duct ligation; c-FLIP_L_: cellular FLICE (FADD-like IL-1*β*-converting enzyme)-inhibitory protein (isoform L); c-FLIP_S_: cellular FLICE- (FADD-like IL-1*β*-converting enzyme-) inhibitory protein (isoform S); CCl_4_: carbon tetrachloride; CFSC: hepatic stellate cell line; DMN: dimethylnitrosamine; ERK: extracellular signal-regulated kinases; FAK: focal adhesion kinase; H_2_O_2_: hydrogen peroxide; HSC: hepatic stellate cells; HSC-T6: immortalized rat liver stellate cell line; I*κ*B-*α*: inhibitor of nuclear factor kappaB alpha; JNK: c-Jun N-terminal kinases; MMP: mitochondrial membrane potential; NF-*κ*B: nuclear factor kappaB; NF-*κ*B p65: p65 subunit of nuclear factor kappaB; p-ERK: phosphorylated extracellular signal-regulated kinases; p-FAK: phosphorylated focal adhesion kinase; PARP: poly ADP ribose polymerase; PCR-HSC: primary cultured rat hepatic stellate cells; PDGF-BB: platelet derived growth factor-BB; PI-3K/AKT: phosphatidylinositide 3-kinases/protein kinase B; PPAR-*γ*: peroxisome proliferator-activated receptor gamma; SD: Sprague-Dawley; TGF-*β*1: transforming growth factor beta 1; T-HSC/Cl-6: rat hepatic stellate cells transformed by simian virus 40; TNF-*α*: tumor necrosis factor alpha; XIAP: X-linked inhibitor of apoptosis protein.

**Table 2:** Mechanisms of protection of hepatocytes from apoptosis by medicinal plants.

Plants	Bioactive compounds and/or extracts	Types of study	Cell lines/animals used	Apoptosis inducers	Mechanisms of protection of hepatocytes from apoptosis
*C. longa *	Curcumin	I [55]	PCRH	Ethanol	↓LPO, ↓cytochrome *c* release
		I [56]	PCRH	FeNTA	↓Bcl-2, ↓Bcl-XL, ↓ROS, ↓caspase-3 and ↓NF-*κ*B
		I [57]	PCRH	HFFAs	↓ROS, ↑ATP, ↓PEPCK, ↓G6Pase, ↑mtDNA copy number, ↑PGC1*α*, ↑NRF1, ↑Tfam, ↑MMP and ↓NF-*κ*B p65

*S. marianum *	Silymarin	I [58]	PCRH	t-BHP	↓NOS-2 and ↓HO-1

*G. biloba *	*G. biloba* extract	II [59]	Rats	CCl_4_	↓LPO
	*G. biloba* extract	II [60]	SD rats	Ethanol	↑GSH, ↓LPO, ↑SOD, ↑GPx, ↑CAT and ↑HO-1
	*G. biloba *	II [61]	Wistar rats	^ 99m^Tc	↓P53/Bcl-2 ratio and ↓LPO

*S. miltiorrhiza *	Tanshinone IIA	I [62]	PCRH	LPS and ethanol	↓ROS, ↓RNS, ↓fatty acid synthesis, ↑fatty acid oxidation, ↓SCD1 and ↑RXR-*α*
		II [63]	Kunming mice	TAA	↓IGFBP7
		I [64]	PCRH	CCl_4_	↑MMP
	Danshen	II [65]	Kunming mice	Iron dextran	↓LPO, ↑GPx and ↑SOD
	PF2401-SF, tanshinone II, tanshinone IIA, cryptotanshinone	I [66]	PCRH	GDCD	↓ROS, ↓JNK phosphorylation and ↓p38 phosphorylation
	PF2401-SF and cryptotanshinone	I [67]	PCRH	LPS and ethanol	↓lipid accumulation and activation, ↓SREBP1 nuclear translocation
	Extract of *S. miltiorrhiza *	I [68]	SD rats	BDL	↑p53 cytoplasmic sequestration, ↓Bax and ↑Bcl-2
	salvianolic acid B	I, II [69]	Mice and HL-7702	Death receptor (I) and LPS + D-GalN (II)	↓TNFR1, ↑Bcl-2, ↓cytochrome *c* release and ↓caspase-3

*G. glabra *	Glycyrrhizin	I [70]	PCRH	t-BHP	↑GSH, ↓ROS, ↑SOD, ↓LPO, ↑MMP, ↓cytochrome *c* release, ↓caspase-3 and ↓caspase-9
		II [71]	SD rats	CCl_4_	↓caspase-3, ↓p53, ↓Bax/Bcl-2 ratio, ↓caspase-9, ↓Smac, ↓cytochrome *c* release and ↓Smac release
		I [72]	PCRH	BCG vaccine + LPS	↓NO and ↓ICAM-1
		II [73]	Wistar rats	LPS	↓caspase-3 and ↓cytochrome *c* release
		II [74]	Balb/c mice	LPS + D-GalN	↓IL-18
		I [75]	Huh-BAT	HMGB1	↓cytochrome *c* release and ↓p38 activation
	18*β*-glycyrrhetinic acid	I [76]	PCRH	GDCD	↓ROS, ↓caspase-3, ↓caspase-9, ↓caspase-10, ↓PARP cleavage, ↓JNK, ↑MMP and ↓cytochrome *c* release
		I, II [77]	HepG2 and SD rats	HFFAs (I) and HFD (II)	Stabilize lysosomal membrane, ↓cathepsin B, ↓cytochrome *c* release and ↓oxidative stress

*S. baicalensis *	Baicalin	I, II [78]	PCRH and Balb/c mice	TNF-*α* + (Act D) (I) and Con A (II)	↓TNF-*α*, ↓IFN-*γ*, ↓IL-6, ↓MPO, ↓LPO and ↓SOD

*Phyllanthus* species	35 kD* P. niruri* protein	I [79]	PCMH	FeSO_4_	↑GSH, ↓GSSG, ↓SOD, ↓CAT, ↓GST, ↓GR, ↓GPx, ↓protein carbonylation, ↓LPO, ↑MMP, ↓cytochrome *c* release, ↓caspase, ↓PARP cleavage, ↑PI3k/Akt, ↓NF-*κ*B phosphorylation, ↓MAPK and ↓ERK
	Geraniin and amariin from *P. amarus *	II [80]	Cultured liver slices of mice	Ethanol	↓LPO, ↓protein carbonylation, ↓CAT, ↓SOD, ↑GPx, ↑GR, ↓PARP cleavage, ↓Bax and ↑Bcl-2
	Protein from *P. niruri *	I [81]	PCMH	t-BHP	↑SOD ↑GSH/GSSG ratio, ↑MMP, ↓Bax, ↑Bcl-2, ↓caspase-3, ↓caspase-9 and ↓cytochrome *c* release

*B. aristata *	Berberine	I [82]	L02	H_2_O_2_	↓caspase-3, ↓PARP, ↓FasL, ↓Bim and ↑SIRT1

*P. kurroa *	Picroside II	II [83]	Kunming mice	CCl_4_, D-GalN and AP	↓LPO, ↑SOD, ↑GPx, ↑ATPase, ↓swelling extent of mitochondria
		I, II [84]	PCRH and Kunming mice	TNF-*α* + Act D (I) and LPS + D-GalN	↓LPO, ↑SOD, ↑Bcl-2 and ↓Bax

*Ginseng *species	*Ginseng* extract from *P. ginseng* Meyer	II [85]	SD rats	AFB_1_	↑SOD, ↑CAT, ↑GPx and ↓LPO

*A. paniculata *	Andrographolide	II [86]	C57BL/6 mice	Con A	↓LDH, ↓MPO, ↓COX2, ↓Glut1, ↓HIF-1*α*, ↓HO-1, ↑SOD1, ↓iNOS and ↓TNF-*α*

Abbreviations: ↑: inductor effect, ↓: inhibitor effect; I: *in vitro*; II: *in vivo*; ^99m^Tc: technetium ^99m^Tc; Act D: actinone D; AFB_1_: aflatoxin B_1_; AP: acetaminophen; ATP: adenosine triphosphate; ATPase: adenosinetriphosphatase; BDL: bile duct ligation; Bim: Bcl-2-interacting mediator; CAT: catalase; CCl_4_: carbon tetrachloride; Con A: concanavalin A; COX2: cyclooxygenase 2; D-GalN: D-galactosamine; FasL: Fas ligand; FeNTA: ferric nitrilotriacetate; FeSO_4_: iron (II) sulfate; G6Pase: glucose-6-phosphatase; GDCD: glycochenodeoxycholic acid; Glut1: glucose transporter 1; GPx: gluthatione peroxidase; GR: glutathione reductase; GSH: glutathione; GSSG: glutathione disulfide; GST: glutathione S-transferase; H_2_O_2_: hydrogen peroxide; HFD: high fat diet; HFFAs: high free fatty acids; HIF-1*α*: hypoxia-inducible factor 1-alpha; HMGB1: high-mobility group box 1; HO-1: heme oxygenase 1; ICAM-1: intercelular adhesion molecule 1; IFN-*γ*: interferon-gamma; IGFBP7: insulin-like growth factor binding protein 7; IL-6: interleukine-6; IL-18: interleukine-18; iNOS: inducible nitric oxide synthase; JNK: c-Jun-NH2-terminal kinase; LDH: lactate dehydrogenase; LPO: lipid peroxidation; LPS: lipopolysaccharide; MAPK: mitogen activated protein kinases; MMP: mitochondrial membrane potential; MPO: myeloperoxidase; mtDNA: mitochondrial DNA; NF-*κ*B: nuclear factor kappaB; NO: nitric oxide; NOS-2: nitric oxide synthase 2; NRF1: nuclear respiratory factor 1; PARP: poly ADP ribose polymerase; PCMH: primary cultured mouse hepatocytes; PCRH: primary cultured rat hepatocytes; PEPCK: phosphoenol pyruvate carboxykinase; PGC1*α*: peroxisome proliferator-activated receptor gamma coactivator 1 alpha; PI3k/Akt: phosphatidylinositide 3-kinases/protein kinase B; RNS: reactive nitrosative species; ROS: reactive oxygen species; RXR-*α*: retinoid-X receptor-alpha; SCD1: stearoyl-CoA desaturase-1; SD: Sprague-Dawley; SIRT1: sirtuin 1; Smac: second mitochondria derived activator of caspases; SOD: superoxide dismutase; SREBP1: sterol regulatory element binding protein-1; t-BHP: tert-butylhydroperoxide; TAA: thioacetamide; Tfam: mitocondrial transcription factor A; TNF-*α*: tumor necrosis factor alpha; TNFR1: tumor necrosis factor alpha receptor type 1.

## References

[1] Mormone E., George J., Nieto N. (2011). Molecular pathogenesis of hepatic fibrosis and current therapeutic approaches. *Chemico-Biological Interactions*.

[2] Ellis E. L., Mann D. A. (2012). Clinical evidence for the regression of liver fibrosis. *Journal of Hepatology*.

[3] Sánchez-Valle V., Chávez-Tapia N. C., Uribe M., Méndez-Sánchez N. (2012). Role of oxidative stress and molecular changes in liver fibrosis: a review. *Current Medicinal Chemistry*.

[4] Friedman S. L. (2003). Liver fibrosis—from bench to bedside. *Journal of Hepatology*.

[5] Poynard T., Mathurin P., Lai C.-L., Guyader D., Poupon R., Tainturier M.-H., Myers R. P., Muntenau M., Ratziu V., Manns M., Vogel A., Capron F., Chedid A., Bedossa P. (2003). A comparison of fibrosis progression in chronic liver diseases. *Journal of Hepatology*.

[6] Lim Y.-S., Kim W. R. (2008). The global impact of hepatic fibrosis and end-stage liver disease. *Clinics in Liver Disease*.

[7] Reeves H. L., Friedman S. L. (2002). Activation of hepatic stellate cells—a key issue in liver fibrosis. *Frontiers in Bioscience*.

[8] Henderson N. C., Iredale J. P. (2007). Liver fibrosis: cellular mechanisms of progression and resolution. *Clinical Science*.

[9] Duval F., Moreno-Cuevas J. E., González-Garza M. T., Rodríguez-Montalvo C., Cruz-Vega D. E. Liver fibrosis and mechanisms of the protective action of medicinal plants—targeting hepatic stellate cell activation and extracellular matrix deposition.

[10] Friedman S. L. (2008). Hepatic stellate cells: protean, multifunctional, and enigmatic cells of the liver. *Physiological Reviews*.

[11] Friedman S. L. (2008). Mechanisms of hepatic fibrogenesis. *Gastroenterology*.

[12] Mann D. A., Marra F. (2010). Fibrogenic signalling in hepatic stellate cells. *Journal of Hepatology*.

[13] Rockey D. C. (2013). Translating an understanding of the pathogenesis of hepatic fibrosis to novel therapies. *Clinical Gastroenterology and Hepatology*.

[14] Rockey D. C. (2008). Current and future anti-fibrotic therapies for chronic liver disease. *Clinics in Liver Disease*.

[15] Wells R. G. (2008). Cellular sources of extracellular matrix in hepatic fibrosis. *Clinics in Liver Disease*.

[16] Pradere J.-P., Kluwe J., de Minicis S., Jiao J.-J., Gwak G.-Y., Dapito D. H., Jang M.-K., Guenther N. D., Mederacke I., Friedman R., Dragomir A.-C., Aloman C., Schwabe R. F. (2013). Hepatic macrophages but not dendritic cells contribute to liver fibrosis by promoting the survival of activated hepatic stellate cells in mice. *Hepatology*.

[17] Oakley F., Meso M., Iredale J. P., Green K., Marek C. J., Zhou X., May M. J., Millward-Sadler H., Wright M. C., Mann D. A. (2005). Inhibition of inhibitor of *κ*b kinases stimulates hepatic stellate cell apoptosis and accelerated recovery from rat liver fibrosis. *Gastroenterology*.

[18] Elsharkawy A. M., Oakley F., Mann D. A. (2005). The role and regulation of hepatic stellate cell apoptosis in reversal of liver fibrosis. *Apoptosis*.

[19] Iredale J. P., Benyon R. C., Pickering J., McCullen M., Northrop M., Pawley S., Hovell C., Arthur M. J. P. (1998). Mechanisms of spontaneous resolution of rat liver fibrosis: hepatic stellate cell apoptosis and reduced hepatic expression of metalloproteinase inhibitors. *The Journal of Clinical Investigation*.

[20] Murphy F. R., Issa R., Zhou X., Ratnarajah S., Nagase H., Arthur M. J. P., Benyon C., Iredale J. P. (2002). Inhibition of apoptosis of activated hepatic stellate cells by tissue inhibitor of metalloproteinase-1 is mediated via effects on matrix metalloproteinase inhibition. Implications for reversibility of liver fibrosis. *The Journal of Biological Chemistry*.

[21] Fallowfield J. A. (2011). Therapeutic targets in liver fibrosis. *The American Journal of Physiology: Gastrointestinal and Liver Physiology*.

[22] Luo Y.-J., Yu J.-P., Shi Z.-H., Wang L. (2004). Ginkgo biloba extract reverses CCl4-induced liver fibrosis in rats. *World Journal of Gastroenterology*.

[23] Qu Y., Chen W.-H., Zong L., Xu M.-Y., Lu L.-G. (2012). 18*α*-Glycyrrhizin induces apoptosis and suppresses activation of rat hepatic stellate cells. *Medical Science Monitor*.

[24] Li X., Peng X.-D., Zhang W.-L., Dai L.-L. (2008). Inhibiting effects of denshensu, baicalin, astragalus and Panax notoginseng saponins on hepatic fibrosis and their possible mechanisms. *Zhonghua Gan Zang Bing Za Zhi*.

[25] Xu J., Fu Y., Chen A. (2003). Activation of peroxisome proliferator-activated receptor-*γ* contributes to the inhibitory effects of curcumin on rat hepatic stellate cell growth. *The American Journal of Physiology—Gastrointestinal and Liver Physiology*.

[26] Zheng S., Chen A. (2004). Activation of PPARγ is required for curcumin to induce apoptosis and to inhibit the expression of extracellular matrix genes in hepatic stellate cells in vitro. *Biochemical Journal*.

[27] Zheng S., Chen A. (2007). Disruption of transforming growth factor-*β* signaling by curcumin induces gene expression of peroxisome proliferator-activated receptor-*γ* in rat hepatic stellate cells. *The American Journal of Physiology: Gastrointestinal and Liver Physiology*.

[28] Zhou Y., Zheng S., Lin J., Zhang Q.-J., Chen A. (2007). The interruption of the PDGF and EGF signaling pathways by curcumin stimulates gene expression of PPAR*γ* in rat activated hepatic stellate cell in vitro. *Laboratory Investigation*.

[29] Cheng Y., Ping J., Xu L.-M. (2007). Effects of curcumin on peroxisome proliferator-activated receptor *γ* expression and nuclear translocation/redistribution in culture-activated rat hepatic stellate cells. *Chinese Medical Journal*.

[30] Priya S., Sudhakaran P. R. (2008). Curcumin-induced recovery from hepatic injury involves induction of apoptosis of activated hepatic stellate cells. *Indian Journal of Biochemistry and Biophysics*.

[31] Lin Y.-L., Lin C.-Y., Chi N.-W., Huang Y.-T. (2009). Study on antifibrotic effects of curcumin in rat hepatic stellate cells. *Phytotherapy Research*.

[32] Shin H. W., Park S. Y., Lee K. B., Jang J.-J. (2009). Down-regulation of Wnt signaling during apoptosis of human hepatic stellate cells. *Hepato-Gastroenterology*.

[33] Zhang X.-L., Liu L., Jiang H.-Q. (2002). Salvia miltiorrhiza monomer IH764-3 induces hepatic stellate cell apoptosis via caspase-3 activation. *World Journal of Gastroenterology*.

[34] Fang S.-M., Li C.-S., An J.-Y., Dun Z.-N., Yao D.-M., Liu L., Zhang X.-L. (2011). The role of extracellular signal-regulated kinase in induction of apoptosis with salvia miltiorrhiza monomer IH764-3 in hepatic stellate cells. *Zhongguo Ying Yong Sheng Li Xue Za Zhi*.

[35] Liu L., Wei J., Huo X., Fang S., Yao D., Gao J., Jiang H., Zhang X. (2012). The Salvia miltiorrhiza monomer IH764-3 induces apoptosis of hepatic stellate cells in vivo in a bile duct ligation-induced model of liver fibrosis. *Molecular Medicine Reports*.

[36] Kim J. Y., Kim K. M., Nan J.-X., Zhao Y. Z., Park P.-H., Lee S. J., Sohn D. H. (2003). Induction of apoptosis by tanshinone I via cytochrome c release in activated hepatic stellate cells. *Pharmacology and Toxicology*.

[37] Che X.-H., Park E.-J., Zhao Y.-Z., Kim W.-H., Sohn D. H. (2010). Tanshinone II A induces apoptosis and s phase cell cycle arrest in activated rat hepatic stellate cells. *Basic and Clinical Pharmacology and Toxicology*.

[38] Pan T.-L., Wang P.-W. (2012). Explore the molecular mechanism of apoptosis induced by tanshinone IIA on activated rat hepatic stellate cells. *Evidence-Based Complementary and Alternative Medicine*.

[39] Lin Y.-L., Lee T.-F., Huang Y.-J., Huang Y.-T. (2006). Antiproliferative effect of salvianolic acid A on rat hepatic stellate cells. *Journal of Pharmacy and Pharmacology*.

[40] Parajuli D. R., Park E.-J., Che X.-H. (2013). PF2401-SF, standardized fraction of *Salvia miltiorrhiza*, induces apoptosis of activated hepatic stellate cells *in vitro* and *in vivo*. *Molecules*.

[41] Chor S. Y., Hui A. Y., To K. F., Chan K. K., Go Y. Y., Chan H. L. Y., Leung W. K., Sung J. J. Y. (2005). Anti-proliferative and pro-apoptotic effects of herbal medicine on hepatic stellate cell. *Journal of Ethnopharmacology*.

[42] Chen M. F., Huang C. C., Liu P. S., Chen C. H., Shiu L. Y. (2013). Saikosaponin a and saikosaponin d inhibit proliferation and migratory activity of rat HSC-T6 cells. *Journal of Medicinal Food*.

[43] Park E.-J., Zhao Y.-Z., Kim J., Sohn D. H. (2006). A ginsenoside metabolite, 20-O-*β*-D-glucopyranosyl-20(S)- protopanaxadiol, triggers apoptosis in activated rat hepatic stellate cells via caspase-3 activation. *Planta Medica*.

[44] Wu Y. L., Wan Y., Jin X. J., Ouyang B. Q., Bai T., Zhao Y. Q., Nan J. X. (2011). 25-OCH3-PPD induces the apoptosis of activated t-HSC/Cl-6 cells via c-FLIP-mediated NF-*κ*B activation. *Chemico-Biological Interactions*.

[45] Malhi H., Gores G. J. (2008). Cellular and molecular mechanisms of liver injury. *Gastroenterology*.

[46] Zhan S.-S., Jiang J. X., Wu J., Halsted C., Friedman S. L., Zern M. A., Torok N. J. (2006). Phagocytosis of apoptotic bodies by hepatic stellate cells induces NADPH oxidase and is associated with liver fibrosis in vivo. *Hepatology*.

[47] Canbay A., Friedman S., Gores G. J. (2004). Apoptosis: the nexus of liver injury and fibrosis. *Hepatology*.

[48] Canbay A., Taimr P., Torok N., Higuchi H., Friedman S., Gores G. J. (2003). Apoptotic body engulfment by a human stellate cell line is profibrogenic. *Laboratory Investigation*.

[49] Mehal W., Imaeda A. (2010). Cell death and fibrogenesis. *Seminars in Liver Disease*.

[50] Jiang J. X., Venugopal S., Serizawa N., Chen X., Scott F., Li Y., Adamson R., Devaraj S., Shah V., Gershwin M. E., Friedman S. L., Török N. J. (2010). Reduced nicotinamide adenine dinucleotide phosphate oxidase 2 plays a key role in stellate cell activation and liver fibrogenesis in vivo. *Gastroenterology*.

[51] Watanabe A., Hashmi A., Gomes D. A., Town T., Badou A., Flavell R. A., Mehal W. Z. (2007). Apoptotic hepatocyte DNA inhibits hepatic stellate cell chemotaxis via toll-like receptor 9. *Hepatology*.

[52] Sohail M. A., Hashmi A. Z., Hakim W., Watanabe A., Zipprich A., Groszmann R. J., Dranoff J. A., Torok N. J., Mehal W. Z. (2009). Adenosine induces loss of actin stress fibers and inhibits contraction in hepatic stellate cells via Rho inhibition. *Hepatology*.

[53] Jiang J. X., Mikami K., Venugopal S., Li Y., Török N. J. (2009). Apoptotic body engulfment by hepatic stellate cells promotes their survival by the JAK/STAT and Akt/NF-*κ*B-dependent pathways. *Journal of Hepatology*.

[54] Jiang J. X., Chen X., Serizawa N., Szyndralewiez C., Page P., Schröder K., Brandes R. P., Devaraj S., Török N. J. (2012). Liver fibrosis and hepatocyte apoptosis are attenuated by GKT137831, a novel NOX4/NOX1 inhibitor in vivo. *Free Radical Biology & Medicine*.

[55] Ghoneim A. I. (2009). Effects of curcumin on ethanol-induced hepatocyte necrosis and apoptosis: implication of lipid peroxidation and cytochrome c. *Naunyn-Schmiedeberg's Archives of Pharmacology*.

[56] Qian J.-J., Zhai X.-G., Niu M.-H., Zhou Q., Zhou Y.-J. (2012). Curcumin inhibits iron overload-induced hepatocytic apoptosis and nuclear factor-*κ*B activity. *National Medical Journal of China*.

[57] Kuo J. J., Chang H. H., Tsai T. H., Lee T. Y. (2012). Curcumin ameliorates mitochondrial dysfunction associated with inhibition of gluconeogenesis in free fatty acid-mediated hepatic lipoapoptosis. *International Journal of Molecular Medicine*.

[58] Černý D., Canová N. K., Martínek J., Hořínek A., Kmoníčková E., Zídek Z., Farghali H. (2009). Effects of resveratrol pretreatment on tert-butylhydroperoxide induced hepatocyte toxicity in immobilized perifused hepatocytes: involvement of inducible nitric oxide synthase and hemoxygenase-1. *Nitric Oxide—Biology and Chemistry*.

[59] Ozenirler S., Dincer S., Akyol G., Ozogul C., Oz E. (1997). The protective effect of Ginkgo biloba extract on CCI4-induced hepatic damage. *Acta Physiologica Hungarica*.

[60] Yao P., Li K., Song F., Zhou S., Sun X., Zhang X., Nüssler A. K., Liu L. (2007). Heme oxygenase-1 upregulated by Ginkgo biloba extract: potential protection against ethanol-induced oxidative liver damage. *Food and Chemical Toxicology*.

[61] Raafat B. M., Saleh A., Shafaa M. W., Khedr M., Ghafaar A. A. (2013). Ginkgo biloba and *Angelica archangelica* bring back an impartial hepatic apoptotic to anti-apoptotic protein ratio after exposure to technetium ^99m^Tc. *Toxicology and Industrial Health*.

[62] Yin H.-Q., Kim Y.-S., Choi Y.-J., Kim Y.-C., Sohn D.-H., Ryu S.-Y., Lee B.-H. (2008). Effects of tanshinone IIA on the hepatotoxicity and gene expression involved in alcoholic liver disease. *Archives of Pharmacal Research*.

[63] Sun R.-F., Liu L.-X., Zhang H.-Y. (2009). Effect of tanshinone II on hepatic fibrosis in mice. *Zhongguo Zhong Xi Yi Jie He Za Zhi*.

[64] Zhu B., Zhai Q., Yu B. (2010). Tanshinone IIA protects rat primary hepatocytes against carbon tetrachloride toxicity via inhibiting mitochondria permeability transition. *Pharmaceutical Biology*.

[65] Gao Y., Wang N., Zhang Y., Ma Z., Guan P., Ma J., Zhang Y., Zhang X., Wang J., Zhang J., Chu L. (2013). Mechanism of protective effects of Danshen against iron overload-induced injury in mice. *Journal of Ethnopharmacology*.

[66] Park E. J., Zhao Y. Z., Kim Y. C., Sohn D. H. (2007). PF2401-SF, standardized fraction of *Salvia miltiorrhiza* and its constituents, tanshinone I, tanshinone IIA, and cryptotanshinone, protect primary cultured rat hepatocytes from bile acid-induced apoptosis by inhibiting JNK phosphorylation. *Food and Chemical Toxicology*.

[67] Yin H.-Q., Choi Y.-J., Kim Y.-C., Sohn D.-H., Ryu S.-Y., Lee B.-H. (2009). Salvia miltiorrhiza Bunge and its active component cryptotanshinone protects primary cultured rat hepatocytes from acute ethanol-induced cytotoxicity and fatty infiltration. *Food and Chemical Toxicology*.

[68] Oh S.-H., Nan J.-X., Sohn D.-H., Kim Y.-C., Lee B.-H. (2002). Salvia miltiorrhiza inhibits biliary obstruction-induced hepatocyte apoptosis by cytoplasmic sequestration of p53. *Toxicology and Applied Pharmacology*.

[69] Yan X., Zhou T., Tao Y., Wang Q., Liu P., Liu C. (2010). Salvianolic acid B attenuates hepatocyte apoptosis by regulating mediators in death receptor and mitochondrial pathways. *Experimental Biology and Medicine*.

[70] Tripathi M., Singh B. K., Kakkar P. (2009). Glycyrrhizic acid modulates t-BHP induced apoptosis in primary rat hepatocytes. *Food and Chemical Toxicology*.

[71] Guo X.-L., Liang B., Wang X.-W., Fan F.-G., Jin J., Lan R., Yang J.-H., Wang X.-C., Jin L., Cao Q. (2013). Glycyrrhizic acid attenuates CCl4-induced hepatocyte apoptosis in rats via a p53-mediated pathway. *World Journal of Gastroenterology*.

[72] Zheng Q.-Z., Lou Y.-J. (2003). Pathologic characteristics of immunologic injury in primary cultured rat hepatocytes and protective effect of glycyrrhizin in vitro. *Acta Pharmacologica Sinica*.

[73] Tang B., Qiao H., Meng F., Sun X. (2007). Glycyrrhizin attenuates endotoxin-induced acute liver injury after partial hepatectomy in rats. *Brazilian Journal of Medical and Biological Research*.

[74] Ikeda T., Abe K., Kuroda N., Kida Y., Inoue H., Wake K., Morito M., Sato T. (2008). The inhibition of apoptosis by glycyrrhizin in hepatic injury induced by injection of lipopolysaccharide/D-galactosamine in mice. *Archives of Histology and Cytology*.

[75] Gwak G.-Y., Moon T. G., Lee D. H., Yoo B. C. (2012). Glycyrrhizin attenuates HMGB1-induced hepatocyte apoptosis by inhibiting the p38-dependent mitochondrial pathway. *World Journal of Gastroenterology*.

[76] Gumpricht E., Dahl R., Devereaux M. W., Sokol R. J. (2005). Licorice compounds glycyrrhizin and 18*β*-glycyrrhetinic acid are potent modulators of bile acid-induced cytotoxicity in rat hepatocytes. *Journal of Biological Chemistry*.

[77] Wu X., Zhang L., Gurley E., Studer E., Shang J., Wang T., Wang C., Yan M., Jiang Z., Hylemon P. B., Sanyal A. J., Pandak W. M., Zhou H. (2008). Prevention of free fatty acid-induced hepatic lipotoxicity by 18beta-glycyrrhetinic acid through lysosomal and mitochondrial pathways. *Hepatology*.

[78] Liu L.-L., Gong L.-K., Wang H., Xiao Y., Wu X.-F., Zhang Y.-H., Xue X., Qi X.-M., Ren J. (2007). Baicalin protects mouse from Concanavalin A-induced liver injury through inhibition of cytokine production and hepatocyte apoptosis. *Liver International*.

[79] Bhattacharyya S., Pal P. B., Sil P. C. (2013). A 35kD Phyllanthus niruri protein modulates iron mediated oxidative impairment to hepatocytes via the inhibition of ERKs, p38 MAPKs and activation of PI3k/Akt pathway. *Food and Chemical Toxicology*.

[80] Londhe J. S., Devasagayam T. P. A., Foo L. Y., Shastry P., Ghaskadbi S. S. (2012). Geraniin and amariin, ellagitannins from *Phyllanthus amarus*, protect liver cells against ethanol induced cytotoxicity. *Fitoterapia*.

[81] Sarkar M. K., Sil P. C. (2010). Prevention of tertiary butyl hydroperoxide induced oxidative impairment and cell death by a novel antioxidant protein molecule isolated from the herb, Phyllanthus niruri. *Toxicology in Vitro*.

[82] Zhu X., Guo X., Mao G., Gao Z., Wang H., He Q., Li D. (2013). Hepatoprotection of berberine against hydrogen peroxide-induced apoptosis by upregulation of sirtuin 1. *Phytotherapy Research*.

[83] Gao H., Zhou Y.-W. (2005). Anti-lipid peroxidation and protection of liver mitochondria against injuries by picroside II. *World Journal of Gastroenterology*.

[84] Gao H., Zhou Y.-W. (2005). Inhibitory effect of picroside II on hepatocyte apoptosis. *Acta Pharmacologica Sinica*.

[85] Kim Y.-S., Kim Y.-H., Noh J.-R., Cho E.-S., Park J.-H., Son H.-Y. (2011). Protective effect of korean red ginseng against aflatoxin B1-induced hepatotoxicity in rat. *Journal of Ginseng Research*.

[86] Shi G., Zhang Z., Zhang R., Zhang X., Lu Y., Yang J., Zhang D., Zhang Z., Li X., Ning G. (2012). Protective effect of andrographolide against concanavalin A-induced liver injury. *Naunyn-Schmiedeberg's Archives of Pharmacology*.

[87] van den Berg R., Haenen G. R. M. M., van den Berg H., Bast A. (2001). Transcription factor NF-*κ*B as a potential biomarker for oxidative stress. *British Journal of Nutrition*.

[88] van Rossum T. G. J., Vulto A. G., Hop W. C. J., Brouwer J. T., Niesters H. G. M., Schalm S. W. (1999). Intravenous glycyrrhizin for the treatment of chronic hepatitis C: a double-blind, randomized, placebo-controlled phase I/II trial. *Journal of Gastroenterology and Hepatology (Australia)*.

[89] van Rossum T. G. J., Vulto A. G., Hop W. C. J., Schalm S. W. (2001). Glycyrrhizin-induced reduction of ALT in European patients with chronic hepatitis C. *The American Journal of Gastroenterology*.

[90] Orlent H., Hansen B. E., Willems M., Brouwer J. T., Huber R., Kullak-Ublick G. A., Gerken G., Zeuzem S., Nevens F., Tielemans W. C. M., Zondervan P. E., Lagging M., Westin J., Schalm S. W. (2006). Biochemical and histological effects of 26 weeks of glycyrrhizin treatment in chronic hepatitis C: a randomized phase II trial. *Journal of Hepatology*.

[91] Manns M. P., Wedemeyer H., Singer A. (2012). Glycyrrhizin in patients who failed previous interferon alpha-based therapies: biochemical and histological effects after 52 weeks. *Journal of Viral Hepatitis*.

[92] Freedman N. D., Curto T. M., Morishima C., Seeff L. B., Goodman Z. D., Wright E. C., Sinha R., Everhart J. E. (2011). Silymarin use and liver disease progression in the Hepatitis C Antiviral Long-Term Treatment against Cirrhosis trial. *Alimentary Pharmacology and Therapeutics*.

[93] Hawke R. L., Schrieber S. J., Soule T. A., Wen Z., Smith P. C., Reddy K. R., Wahed A. S., Belle S. H., Afdhal N. H., Navarro V. J., Berman J., Liu Q.-Y., Doo E., Fried M. W. (2010). Silymarin ascending multiple oral dosing phase I study in noncirrhotic patients with chronic hepatitis C. *Journal of Clinical Pharmacology*.

[94] Schrieber S. J., Hawke R. L., Wen Z., Smith P. C., Reddy K. R., Wahed A. S., Belle S. H., Afdhal N. H., Navarro V. J., Meyers C. M., Doo E., Fried M. W. (2011). Differences in the disposition of silymarin between patients with nonalcoholic fatty liver disease and chronic hepatitis C. *Drug Metabolism and Disposition*.

[95] Fried M. W., Navarro V. J., Afdhal N., Belle S. H., Wahed A. S., Hawke R. L., Doo E., Meyers C. M., Reddy K. R. (2012). Effect of silymarin (milk thistle) on liver disease in patients with chronic hepatitis C unsuccessfully treated with interferon therapy: a randomized controlled trial. *Journal of the American Medical Association*.

[96] Liu P., Hu Y.-Y., Liu C., Zhu D.-Y., Xue H.-M., Xu Z.-Q., Xu L.-M., Liu C.-H., Gu H.-T., Zhang Z.-Q. (2002). Clinical observation of salvianolic acid B in treatment of liver fibrosis in chronic hepatitis B. *World Journal of Gastroenterology*.

[97] She S.-F., Huang X.-Z., Tong G.-D. (2004). Clinical study on treatment of liver fibrosis by different dosages of Salvia injection. *Zhongguo Zhong Jie He Za Zhi*.

[98] Ye F., Liu Y., Qiu G., Zhao Y., Liu M. (2005). Clinical study on treatment of cirrhosis by different dosages of salvia injection. *Zhong Yao Cai*.

[99] Jin C.-X., Yang J., Sun H.-F. (2006). Comparative study of the clinical effects of salvia miltiorrhiza injection and shengmai injection on chronic hepatitis B. *Zhongguo Zhong Xi Yi Jie He Za Zhi*.

